# Mismatch repair deficiency is associated with specific morphologic features and frequent loss of ARID1A expression in ovarian clear cell carcinoma

**DOI:** 10.1186/s13000-021-01071-w

**Published:** 2021-02-04

**Authors:** Huijuan Ge, Yaoxin Xiao, Guangqi Qin, Yanzi Gu, Xu Cai, Wenhua Jiang, Xiaoyu Tu, Wentao Yang, Rui Bi

**Affiliations:** 1 Department of Pathology, Fudan University Shanghai Cancer Center, Fudan University, 270 Dong An Road, 200032 Shanghai, China; 3 Department of Oncology, Shanghai Medical College, Fudan University, Shanghai, China; 2 Departments of Pathology, Obstetrics and Gynecology Hospital of Fudan University, Shanghai, China

**Keywords:** Ovarian clear cell carcinoma, Deficient MMR (dMMR), Diffuse intratumoral stromal inflammation, MSH2/MSH6, ARID1A

## Abstract

**Background:**

Ovarian clear cell carcinoma (OCCC) is the second subtype of ovarian epithelial carcinoma reported to be closely related to Lynch syndrome (LS). ARID1A mutation is an important pathogenetic mechanism in OCCC that leads to loss of ARID1A expression in approximately half of OCCCs. However, the correlation of MMR status and ARID1A deficiency is unclear. The current study aimed to identify the clinical and histopathological characteristics of OCCC associated with dMMR and to further explore the association between dMMR and ARID1A deficiency.

**Methods:**

A cohort of 176 primary OCCC patients was enrolled and review included histological characteristics (nuclear atypia, necrosis, mitosis, stromal hyalinization, and background precursors) and host inflammatory response (tumor-infiltrating lymphocytes, peritumoral lymphocytes, intratumoral stromal inflammation and plasma cell infiltration). Immunohistochemical staining of MLH1, PMS2, MSH2, MSH6 and ARID1A was performed using tissue microarrays.

**Results:**

dMMR was detected in 10/176 tumors (6 %), followed by MSH2/MSH6 (6/176), MLH1/PMS2 (3/176), and MSH6 (1/176). The average age of patients with dMMR was younger than that of patients with intact MMR (46 y vs. 53 y). Tumors with diffuse intratumoral stromal inflammation remained significantly associated after multivariate analysis. ARID1A expression was absent in 8 patients with dMMR (8/10), which is a significantly higher frequency than that observed in patients with intact MMR (80 % vs. 43.2 %).

**Conclusions:**

Our study indicates that diffuse intratumoral stromal inflammation of OCCCs is associated with dMMR, with loss of MSH2/MSH6 expression being most frequent. dMMR is strongly associated with the loss of ARID1A expression in OCCC.

## Introduction

Lynch syndrome (LS) is an autosomal dominant predisposition toward neoplasia caused predominantly by germline mutations in one of four DNA mismatch repair (MMR) genes, including MLH1, MSH2, MSH6 and PMS2 [1], and rarely by EpCAM (epithelial cell adhesion molecule) deletions, which cause MSH2 promoter methylation and ultimately inactivation [2]. A loss-of-function mutation in the normal allele increases cancer risk [3] and is the most common cause of colon cancer and familial endometrial carcinoma. It has also been associated with an increased risk of other cancers, such as renal pelvis/ureter, stomach, small bowel, and brain cancer [4].

Among gynecologic cancers, endometrial carcinoma is the most common tumor related to LS, followed by ovarian cancer, with cumulative lifetime risks of 21–71 % [5, 6] and 6–12 % [7, 8], respectively. Although endometrial carcinoma surveillance can be effective, the value of ovarian cancer surveillance is still controversial. Ovarian cancer with LS is frequently of the endometrioid/clear cell carcinoma histological type [3, 9, 10]. Ovarian clear cell carcinoma (OCCC) is the second reported subtype of ovarian epithelial carcinoma that is closely related to LS, with a mean age at diagnosis of 55 years, and is strongly associated with endometriosis and adenofibromatous components [11]. Several reports have demonstrated that tumor-infiltrating lymphocytes (TILs) and peritumoral lymphocytes in endometrioid carcinoma indicate deficient MMR (dMMR) [12–14]. However, morphological features indicating dMMR in OCCCs have rarely been described. Although Bennett et al. reported diffuse intratumoral stromal inflammation to be an indicator of dMMR in OCCCs [15], no further research has been performed to confirm this finding.

The *ARID1A* gene encodes the BAF250A protein, and abnormalities in this gene or loss of protein expression have been shown to be key and early molecular events in the progression of OCCC [16, 17]; additionally, *PIK3CA* or *PTEN* alterations appear to be required for this process [18, 19]. Inactivation of the ARID1A subunit is observed in approximately half of all OCCCs [16, 20], although no reports have examined the relationship between ARID1A and MMR expression in OCCCs.

In this study, we aimed to identify the clinical and histopathological characteristics of OCCC associated with dMMR and to further examine the relationship between dMMR and ARID1A expression.

## Materials and Methods

### Patients and tissue samples

A cohort of 176 unselected primary OCCC patients was enrolled at the Department of Pathology, Fudan University Shanghai Cancer Center from 1999 to 2016. Tumor specimens were collected after obtaining written consent from all patients with the approval of the Facility Ethical Committee (Fudan University Cancer Center Ethics Committee, China; approval No. 050432-4-121B, 13 December 2012).

All cases were reviewed, and the diagnosis was confirmed by two pathologists (Dr Ge and Dr Bi). All 176 cases were pure OCCCs, including two cases of synchronous endometrial endometrioid carcinoma. The following clinical data were extracted from the medical history and examination records: age, tumor size, and Federation of Gynecology and Obstetrics (FIGO) stage. All international FIGO stages were reclassified according to the 2014 FIGO guidelines [21].

### Tissue microarray (TMA) construction

 Hematoxylin-eosin (HE)-stained slides were reviewed by Dr Ge and Dr Bi, and an abundant tumor cell area was chosen for TMA construction.

The TMA was prepared using triplicate core samples from 176 OCCCs to achieve a high level of standardization for immunohistochemical analysis [22]. Triplicate 1-mm tissue cores were taken from each targeted donor block, and a receptor block was inserted. Four-micrometer sections were cut and stained with H&E to confirm the histologic diagnosis. Unstained slides were used for immunohistochemical staining.

### Immunohistochemistry

Immunohistochemical staining was performed on TMAs with the Ventana Benchmark XT platform. The following panel of antibodies was used: anti-MLH1 (clone G168-728, Roche), -PMS2 (clone EPR3947, Roche), -MSH2 (clone G219-1129, Roche), -MSH6 (clone 44, Roche), and -ARID1A (clone D2A8U, Roche). According to the published criteria of abnormal expression of MMR and ARID1A [15, 23], both markers were interpreted as loss of expression, whereas no tumor cells were stained.

We chose the recommended guidelines that the immunohistochemistry results be reported in a binary manner, either positive (indicating intact mismatch repair, showing intact nuclear expression in tumor cells) or negative (indicating deficient mismatch repair, showing nuclear expression completely lost in tumor cells) [24, 25] Nuclei of lymphocytes and ovarian stromal cells served as positive internal controls. All staining results were reviewed by the above 2 pathologists. Because immunohistochemical examination was performed using a tissue microarray, when deficient mismatch repair (dMMR) was observed, the immunohistochemistry procedure was repeated on whole-slide sections to avoid heterogeneous expression, such as for MSH6 [26]. Ultimately, MMR protein restaining was performed in 51 instances involving 27 of 176 cases; ARID1A restaining was performed in 15 instances involving 3 cases of dMMR among 135 cases.

### Morphologic assessment

All tumor slides were evaluated by Dr Ge and Dr Bi, who were blinded to the MMR status, and an average of 7 tumor slides per case (range 1–17) were examined. The following features were assessed (in brief) [8].

### Host inflammatory response

**Fig. 1:**
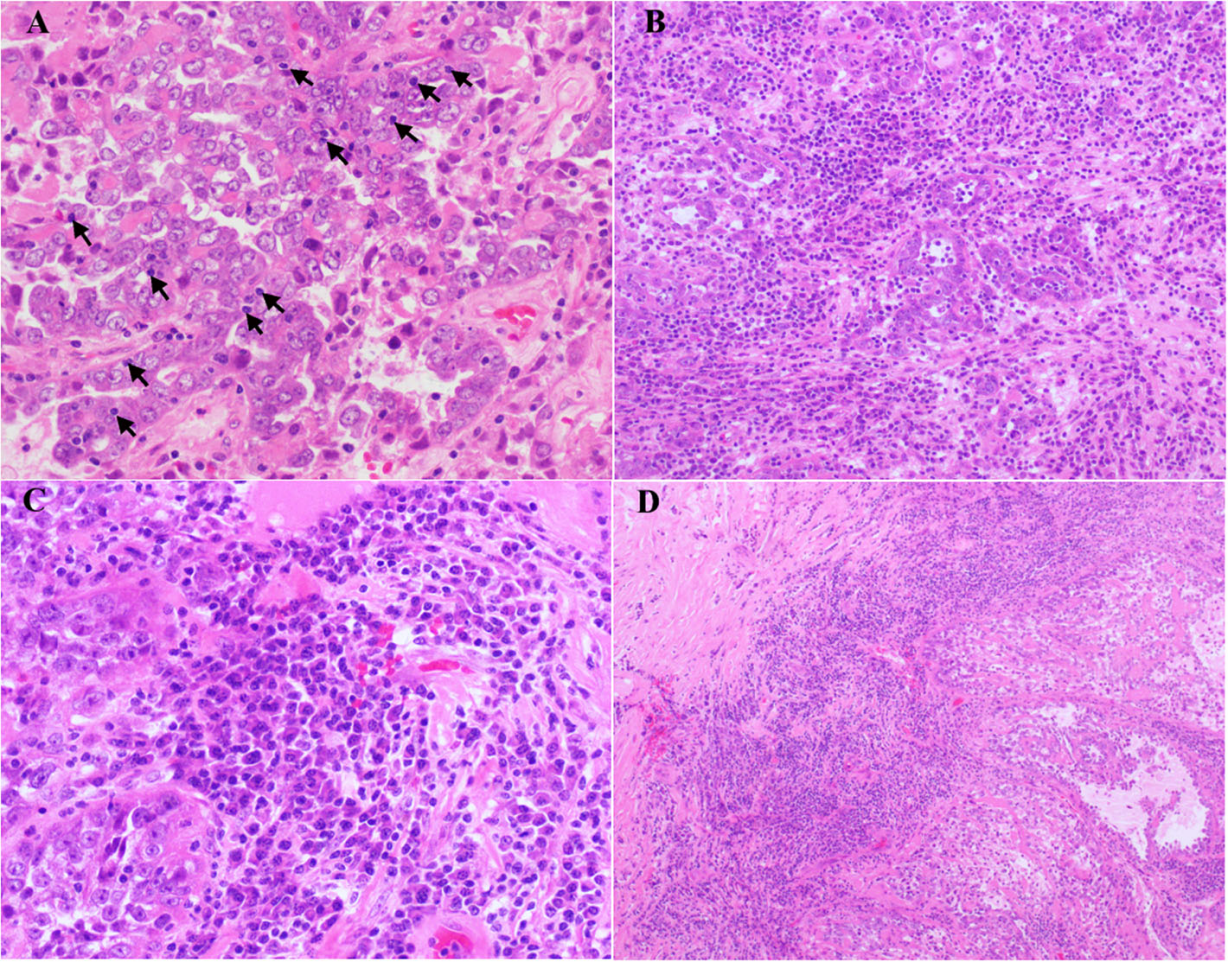
Tumor-infiltrating lymphocytes (TILs) (**a**, arrows) in tumor cells, diffuse intratumoral stromal inflammation (**b**), composed mostly of plasma cells (**c**), and peritumoral lymphocytes (**d**) were observed

1. TILs – defined only as lymphocytes located within the boundary of tumor cells or glands – were counted, especially in regions of high tumor density and minimal stroma. Areas showing the greatest number of lymphocytes at low-power magnification were randomly selected, and the numbers of lymphocytes were counted in 10 high-power microscopic fields (HPFs). Apoptotic bodies and stromal lymphocytes were not counted (Fig. 1a).
2. Intratumoral stromal inflammation – defined as inflammation in the stroma between tumor cells, nests, or glands that is evident at low-power magnification – was graded as diffuse (marked inflammatory infiltrate present in multiple foci or on multiple slides), focal (partially present in the stroma with inflammation) or absent (8). (Fig. 1b). Areas of necrosis and infarction were excluded.
3. Peritumoral lymphocytes – defined as peritumoral lymphocytes present at low-power magnification – were categorized as present or absent (Fig. 1c).
4. Plasma cell infiltration – defined as the percentage of plasma cells in the intratumoral stroma – was classified as < 10 %, 10–50 %, or > 50 % (Fig. 1d).

### Tumor characteristics

1. Background precursor – evaluated based mainly on the benign element adjacent to the tumor in the same section, categorized as a tumor arising as endometriosis, adenofibroma, both, or neither.
2. Architectural pattern – included tubulocystic, papillary, glandular, solid or mixed.
3. Stromal hyalinization – graded as diffuse, focal, or absent.
4. Nuclear atypia – high cytologic grade demonstrating variations of greater than 3 times the nuclear size, highly irregular nuclear contours, striking hyperchromasia, and/or prominent nucleoli. Areas constituting at least 10 % of the tumor were considered high grade; other areas were considered low grade.
5. Specific morphology (signet ring cells) – defined as present or absent.
6. Tumor necrosis – defined as present when there were geographic foci of tumor cell necrosis. Areas of necrosis only within glands or at the tumor’s surface were not included.

### Follow‐up

Overall survival (OS) was defined as the time from the operation to either death or the last follow-up. Disease-free survival (DFS) was defined as the interval from the operation to disease recurrence or the last follow-up. We defined disease recurrence as a consistent elevation in CA125 or detection of a tumor by a clinical examination, including a physical examination and imaging [27]. The patients were followed up until December 2018 by medical records or telephone.

### Statistical analysis

Statistical analysis was conducted using SPSS version 20.0 software (IBM, SPSS Statistics Armonk, NY, and USA). Univariate analysis of categorical variables was performed with the chi-square test and Fisher’s exact test; multivariate analysis was performed with exact logistic regression to identify independent predictors of abnormal MMR expression. Survival analysis was performed using the Kaplan-Meier univariate method. Differences in survival curves were determined with log-rank tests. *P* values < 0.05 were considered statistically significant.

## Results

### Clinicopathological factors

Patients with dMMR were younger than those with intact MMR (ranging from 28 to 45 y, mean 46 y vs. range 22–83 y, mean 53 y, *P* = 0.041). Macroscopically, the tumor size ranged from 6 to 20 cm (mean 11.25 ± 5.5 cm). Tumors occurred on the right side of the ovary in 80 patients (45.4 %) and on the left side of the ovary in 64 (36.4 %); 32 patients (18.2 %) had bilateral tumors. More than half of the cases were stage I/II (66 %, 116 patients), whereas 60 cases were stage III/IV (34 %). In the dMMR group, 2 patients had synchronous malignant tumors (uterus endometrioid carcinoma, grade I-II), and 2 patients had a history of hereditary nonpolyposis colorectal cancer (HNPCC).

### Histological characteristics

Among the morphologic variables evaluated, intratumoral stromal inflammation and plasma cell infiltration were both significantly associated with MMR status. Patients with dMMR and intact MMR exhibited no significant differences in endometriosis, adenofibromatous background, TILs, peritumoral lymphocytes, necrosis, stromal hyalinization, grade or stage (Table 1).

**Table 1:** Pathologic characteristics of clear cell carcinoma and their association with MMR status

		MMR-deficient cases (n = 10)	MMR-intact cases (n = 166)	P
Age(y)				0.078
<50		6 (60 %)	54 (32.5 %)	
≥50		4 (40 %)	112 (67.5 %)	
Median	46		53	0.041
Family history of cancer		2	39	1.0
HNPCC history		2	20	0.362
Precursor Not present Adenofibroma Endometriosis both		4 (40 %) 1 (10 %) 4 (40 %) 1 (10 %)	61 (36.7 %) 22 (13.3 %) 69 (41.6 %) 14 (8.4 %)	0.987
Stromal Hyalinization				0.671
Focal or absent		8 (80 %)	139 (83.7 %)	
Diffuse		2 (20 %)	27 (16.3 %)	
Nuclear Atypia				0.139
High grade		5 (50 %)	43 (25.9 %)	
Low-medium grade		5 (50 %)	123 (74.1 %)	
Signet ring cells				0.603
Not present		10 (100 %)	147 (88.6 %)	
Present		0 (0 %)	19 (11.4 %)	
Peritumoral Lymphocytes				0.127
Not present		7 (70 %)	146 (88 %)	
Present		3 (30 %)	20 (12 %)	
Intratumoral Stromal Inflammation				0.001
Diffuse		5 (50 %)	11 (6.6 %)	
Focal		5 (50 %)	155 (93.4 %)	
Plasma Cell Component				0.037
≤50 %		6 (60 %)	145 (87.3 %)	
>50 %		4 (40 %)	21 (12.7 %)	
Tumor infiltrating Lymphocytes (/10HPF)				0.249
< 40		8 (80 %)	151 (91 %)	
≥ 40		2 (20 %)	15 (9 %)	
Mitosis (/10HPF)		5.1	6.4	0.499
Necrosis				0.300
Not present		3 (30 %)	73 (44 %)	
Present		7 (70 %)	93 (56 %)	
ARID1A (135 cases)				0.027
Negative		8 (80 %)	54 (43.2 %)	
Positive		2 (20 %)	71 (56.8 %)	
Stage				0.462
I/II		6	110	
III-IV		4	56	

### Host inflammatory response

Prominent intratumoral stromal inflammation and > 50 % plasma cells were both more frequent in tumors with dMMR (*P* = 0.001 and *P* = 0.037, respectively), but only diffuse intratumoral stromal inflammation correlated significantly with dMMR in multivariate analysis (*P* = 0.004, Table 2).

**Table 2:** Multinomial logistic regression for morphologic features indicating dMMR in OCCCs

	Relative risk (95 % CI)	*P* value
Intratumoral Stromal Inflammation		
Diffuse vs. focal	0.048 (0.006–0.409)	0.004
Plasma Cell Component		
≤ 50 % vs. >50 %	1.702 (0.187–15.522)	0.632

### Relationship between dMMR and ARID1A expression

**Fig. 2:**
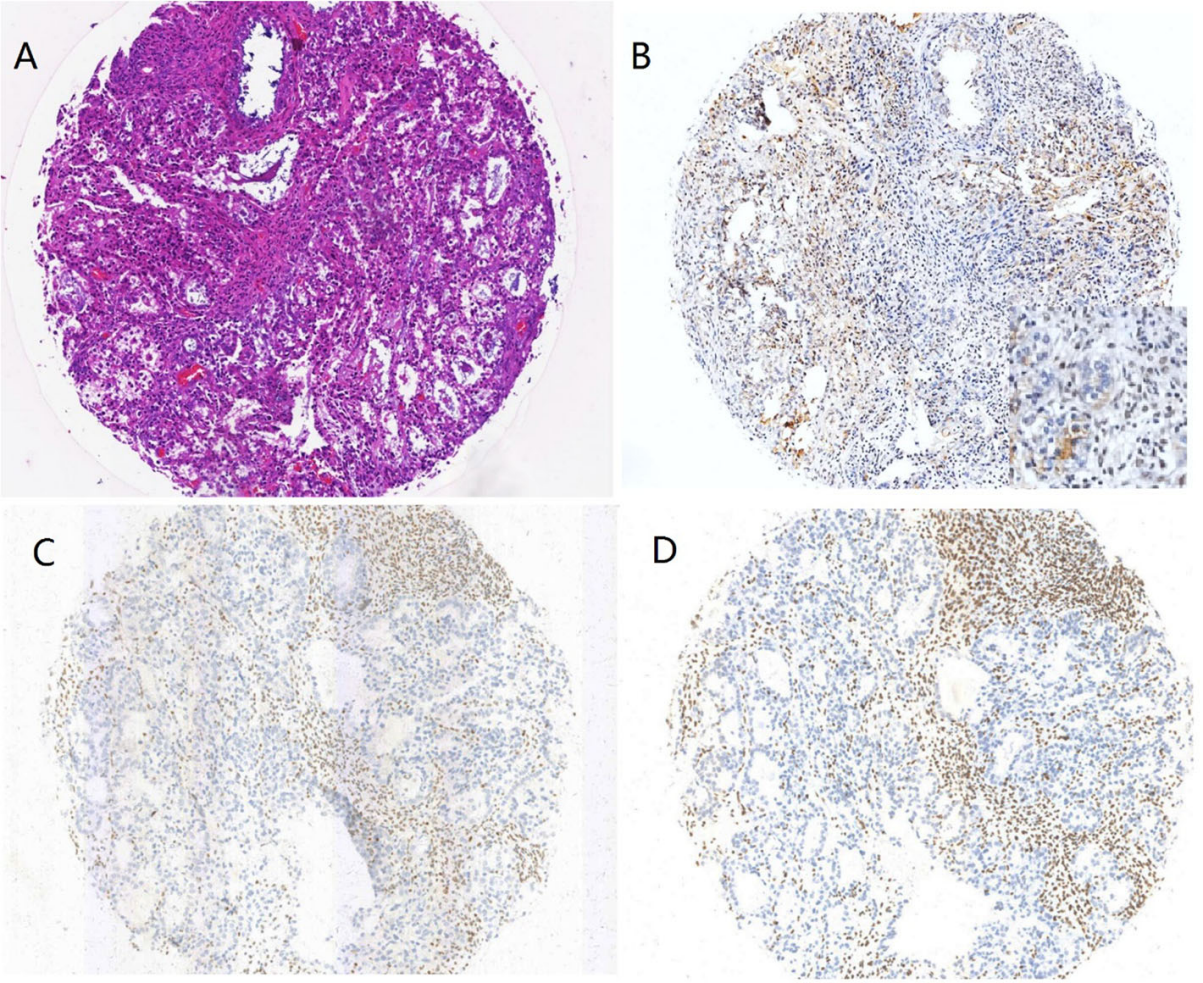
One case (CCC_085) (HE, **a**) showed a synchronous loss of ARID1A (**b**), MSH2 (**c**) and MSH6 (**d**) expression

**Fig. 3:**
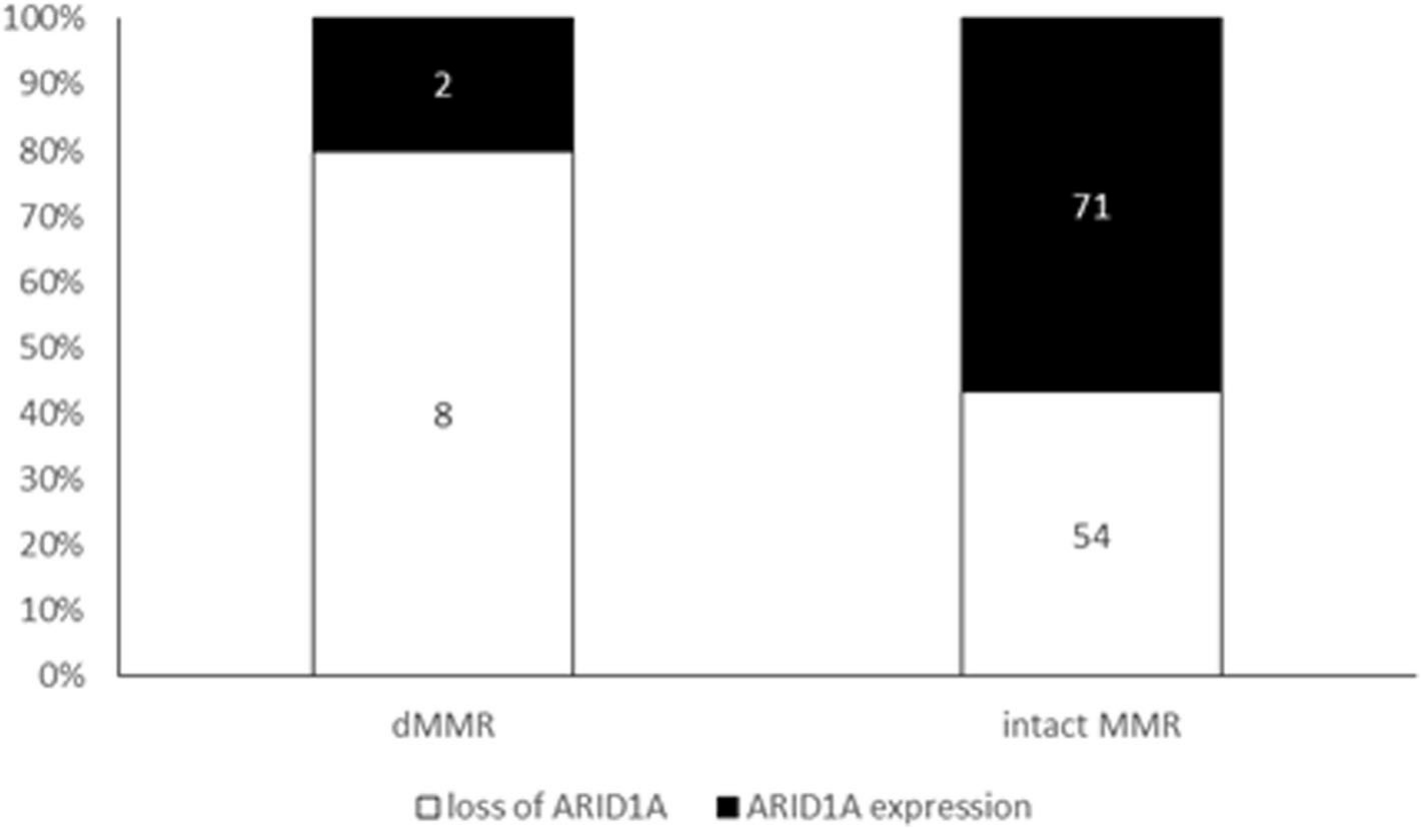
Distribution of ARID1A expression in OCCC. Loss of ARID1A expression was more frequent in dMMR OCCC than in intact MMR OCCC (80 % vs. 43 %, *P* = 0.027)

A total of 176 patients underwent immunohistochemical analysis for MMR, among which only 10/176 (6 %) exhibited dMMR after restaining on the whole slides. The most frequently absent MMR protein was MSH2/MSH6, in 6/10 (60 %) dMMR patients (Fig. 2), followed by MLH1/PMS2 in 3/10 patients (30 %); 1 patient (1/10, 10 %) showed isolated loss of MSH6. In total, ARID1A staining was successful for 135 cases, including 62 with loss of expression (46 %) and 73 with intact expression. Among the 10 dMMR patients, 2 exhibited ARID1A staining, but 8 (80 %) were deficient (Table 3); for patients with intact MMR, 54 (43 %) exhibited loss of ARID1A, and 71 (57 %) showed positive expression. There was a significant frequent loss of ARID1A expression in dMMR OCCC compared to intact MMR OCCC (80 % vs. 43 %, *P* = 0.027, Fig. 3).

**Table 3:** MMR deficient cases with expression of ARID1A

MLH1	PMS2	MSH2	MSH6	ARID1A
0	0	1	1	0
0	0	1	1	0
0	0	1	1	0
1	1	1	0	0
1	1	0	0	0
1	1	0	0	1
1	1	0	0	1
1	1	0	0	0
1	1	0	0	0
1	1	0	0	0

0: MMR deficient; 1: MMR intact

### Follow‐up

Follow-up information was available for 97.2 % (171/176) of patients, and the mean and median follow-up times were 57 and 46.7 months, respectively (range, 0.9-187.7 months). The OS and 5-year OS rates were 119/171 (67.7 %) and 26/171 (71.6 %), respectively. No significant differences in OS or DFS (*P* = 0.920 and *P* = 0.974, respectively) between the dMMR and intact MMR patients were found.

## Discussion

The rate of MMR deficiency has been reported to be 12-13.8 % in a large cohort of ovarian endometrioid carcinoma patients [10, 28] 2 and 2 %-6 % in OCCC patients [10, 15, 29, 30] In our cohort, ten patients with dMMR were observed among 176 OCCC patients (10/176, 6 %), which is a similar frequency to that reported by Bennett et al. for 109 OCCC patients (6/109, 6 %) [15] but higher than the rate of 2.4 % reported by Rambau et al [10]. Based on data from these two previous studies [10, 15] and our series, the dMMR frequency in ovarian cancer differs from that in endometrial carcinoma. The highly frequent deficiency of MSH2/MSH6 or MSH6 expression in ovarian endometrioid carcinoma and OCCC, as opposed to the most MHL1/PMS2 expression deficiency in endometrial carcinoma [14, 31], indicates a high probability of LS. Further, further studies are needed to examine MMR germline mutations and MLH1 promoter hypermethylation in our cohort.

The average age of patients with dMMR was 46 y in our series, with an age range of 28–58 y, which was younger than that of individuals with intact MMR (mean 53 y, range of 22–83 y), and 60 % of patients were younger than 50 y. dMMR was observed in 10 % of all patients under 50 y (6/60, 10 %), which was a higher frequency than that observed in the whole series (6 %). However, the prevalence among our cohort was less than the rate of 17 % reported by Jensen et al [32]. This is comparable with the age at diagnosis in 747 ovarian cancer patients with LS described in a recent review of the literature, with a mean age of 45.3 y (range 19–92 y) [33], and with a recently published cohort study of 53 ovarian cancers in LS with a mean age of 51 y (range 24–70 y) [34]. LS-associated ovarian cancer seems to occur approximately 15–20 y earlier than sporadic ovarian cancer [35]. Interestingly, OCCC synchronous with grade I-II endometrial endometrioid carcinoma, which is probably sporadic but does not completely exclude LS, was noted in 2 of 3 MLH1/PMS2-deficient patients. In addition, synchronous tumors in the ovary and endometrium have been hypothesized to likely be sporadic [36]. However, Martin et al [37] investigated the frequency of MMR protein deficiency in a large series of endometrial mixed endometrioid and clear cell carcinomas detecting it in 27 (27/41, 66 %) cases. Of these 27 MMR-deficient tumors, most (16, 59 %) showed concurrent MLH1 and PMS2 loss. These results are consistent with our findings. The pattern of MMR protein deficiency was identical between the corresponding endometrioid and clear cell components in all MMR protein-deficient cases.

Although several morphologic features are believed to be associated with abnormal MMR in endometrial and colonic carcinomas [12, 14], TILs, as a predictive morphologic feature, are not associated with OCCC or ovarian endometrioid carcinoma [9, 28]. Bennett et al. first presented diffuse intratumoral stromal inflammation and peritumoral lymphocytes as 2 potential features predictive of dMMR in OCCC [15]. In our cohort, we further confirmed that diffuse intratumoral stromal inflammation is an independent feature associated with dMMR status, offering the possibility of economical screening because of the low frequency of dMMR (usually < 10 %) in OCCC.

In one series [38], The lack of an adenofibromatous background was found to be predictive of dMMR in ovarian endometrioid carcinoma, although none of our dMMR OCCC patients showed such a background. Tumor grade, stage, size, endometriosis, and mitotic index were also unrelated to MMR status.

Loss of ARID1A is a frequent event in OCCC, with 35–61 % of patients exhibiting deficiency in ARID1A expression [16, 39–41] We detected loss of ARID1A expression in 46 % of OCCCs, and 6 % of these cases indicated dMMR. It is worth noting that the dMMR patients exhibited a high frequency of loss of ARID1A expression in our cohort (P = 0.027). This is consistent with reports of colorectal carcinoma [42], gastric carcinoma [43], endometrial carcinoma [44, 45] and ovarian endometrioid carcinoma [10], indicating a correlation between dMMR and loss of ARID1A expression. ARID1A mutation is the core mechanism of OCCC and exhibits a correlation with loss of the protein [16, 17]. Moreover, loss of ARID1A is significantly associated with microsatellite instability (MSI), predominantly resulting from sporadic MSI (MLH1 promoter hypermethylation), in endometrial cancer [44]. ARID1A deficiency impairs the DNA damage checkpoint [46], which is targeted by the MSI mechanism of DNA damage in gastric cancer [43], and ARID1A recruits MSH2 to chromatin during DNA replication and promotes MMR [47]. Taken together, these findings may contribute to the association of the loss of ARID1A expression with dMMR.

Although we did not find statistically significant differences in OS and DFS between dMMR and intact MMR patients, the results indicated that the mean survival period was longer in patients with dMMR than in patients with intact MMR (140 months vs. 116 months for OS; 120 months vs. 100 months for DFS). Two factors likely contribute to the lack of difference between the dMMR and intact MMR groups: one is that all-cause survival data rather than disease-specific survival data were investigated, and the other factor may be related to the high percentage of dMMR patients who were lost to follow-up (20 %). Stewart et al. reported two patients who showed loss of MSH2/MSH6 expression and were alive at 160 months and 124 months with stage III/IV OCCCs [48].

## Conclusions

To our knowledge, the current study is the largest series to explore the MMR expression status and the clinicopathologic features of consecutive OCCC cases. We conclude that dMMR occurs in a small proportion of patients but indicates intratumoral stromal inflammation in OCCCs, which correlates with loss of ARID1A expression. Loss of MSH2/MSH6 expression is the most common deficiency pattern.

## Data Availability

The datasets generated and /or analysed during the current study are not publicly available due the institutional review board restricts the use of the datasets to the current study only.

## Abbreviations

OCCC: Ovarian clear cell carcinoma
LS: Lynch syndrome
MMR: Mismatch repair
dMMR: Deficient MMR
TILs: Tumor-infiltrating lymphocytes
TMA: Tissue microarray
HE: Hematoxylin-eosin
HPFs: High-power microscopic fields
OS: Overall survival
DFS: Disease-free survival
HNPCC: Hereditary nonpolyposis colorectal cancer
